# Genome Analysis of *Lactobacillus plantarum* LL441 and Genetic Characterisation of the Locus for the Lantibiotic Plantaricin C

**DOI:** 10.3389/fmicb.2018.01916

**Published:** 2018-08-17

**Authors:** Ana B. Flórez, Baltasar Mayo

**Affiliations:** Departamento de Microbiología y Bioquímica, Instituto de Productos Lácteos de Asturias – Consejo Superior de Investigaciones Científicas, Villaviciosa, Spain

**Keywords:** *Lactobacillus plantarum*, genome sequencing, genome analysis, lactic acid bacteria, adjunct cultures, bacteriocins, lantibiotics, plantaricin C

## Abstract

Bacteriocins are ribosomally synthesized peptides produced by bacteria with antimicrobial activity. The bacteriocins produced by lactic acid bacteria (LAB) may inhibit food-borne pathogens and spoilage organisms, and therefore have potential as natural preservatives. *Lactobacillus plantarum* LL441 produces a lantibiotic bacteriocin known as plantaricin C, a pore-forming antimicrobial peptide containing modified amino acids that inhibits cell wall synthesis by forming a complex with the peptidoglycan precursor lipid II. The present work describes the genome sequencing of *L. plantarum* LL441 and the characterisation of the plantaricin C locus. The draft genome sequence of *L. plantarum* LL441 consisted of 170 contigs and had a total 3,124,603 bp; the GC content was 44.52%. The plantaricin C locus was found in an 18 kbp-long contig, and consisted of six genes organized in an operon-like arrangement. This locus included the bacteriocin structural gene (*plnC*), followed by a gene encoding a LanM-like protein thought to be involved in the maturation of plantaricin C, and four downstream genes encoding ABC-type transporter components, probably belonging to its putative immunity and export machinery. *plnC* encodes a precursor of the bacteriocin, i.e., a 58-amino acid peptide containing a 31-amino acid double-glycine leader peptide and a 27-amino acid core peptide. *In silico* analysis and hybridisation experiments placed the plantaricin C locus to be located on pLL441-1, a large plasmid of *L. plantarum* LL441. Joining up the gaps between the contigs by conventional PCR, sequencing of the amplicons, and sequence assemblage, allowed the complete 55.3 kbp pLL441-1 molecule to be established. A portion of pLL441-1 larger than 34 kbp, which included the plantaricin C region, was identified in a plasmid-derived contig from the *L. plantarum* Nizo 3893 genome. Further, the plantaricin C coding locus (about 8.7 kbp) was shown to share 91% nucleotide identity with a portion of the plasmids pPECL-6 from *Pediococcus claussenii* ATCC BAA-344 and pL11995-4 from *Lactobacillus paracollinoides* TMW 1.1995. Knowledge of the sequence of the plantaricin C coding region will help in studying its molecular components and allow their involvement in bacteriocin synthesis to be investigated, facilitating the use of the bacteriocin or its genetic elements in new biotechnological applications.

## Introduction

*Lactobacillus plantarum* is a lactic acid bacterium (LAB) found in nutritive-rich environments, e.g., plant-, meat-, fish-, and dairy products, as well as animal and human mucosae (Siezen et al., 2010). The remarkable ecological adaptability of *L. plantarum* to such different ecological niches reflects its capacity to ferment a large range of carbohydrates, including monosaccharides, disaccharides, and polysaccharides (Di Cagno et al., 2010; Siezen et al., 2010; Siezen and van Hylckama Vlieg, 2011), from which lactic acid is produced as the majority final metabolite. Some *L. plantarum* strains also produce antimicrobial substances such as H_2_O_2_, organic and fatty acids, and bacteriocins (Arqués et al., 2015). Bacteriocins are ribosomally synthesized, antimicrobial peptides or proteins that inhibit the growth of, or kill, other bacteria (Álvarez-Sieiro et al., 2016). Those produced by LAB species have the potential to inhibit or kill food-borne spoilage and pathogenic bacteria, and might be contemplated as natural preservatives (Perez et al., 2014). They may therefore limit the use of chemicals by the food industry, and reduce the intensity of the heat treatments required in the production of certain foods (Gálvez et al., 2007; Ramu et al., 2015). Recently, bacteriocins have also been reported to have possible therapeutic applications in the clinic, in animal husbandry, and in aquaculture (Bali et al., 2016).

Most bacteriocins are cationic peptides (20–70 amino acids long) that destabilize the integrity of the inner cell envelope, causing membrane potential disruptions and/or the leakage of essential solutes (Perez et al., 2014; Álvarez-Sieiro et al., 2016). Based on the biosynthesis mechanism and biological activity, bacteriocins produced by LAB are currently classified into three major classes: (i) peptides that undergo enzymatic modification during biosynthesis (Class I), (ii) unmodified small peptides (<10 kDa), (Class II), and (iii) unmodified large peptides (>10 kDa) with bacteriolytic or non-lytic mechanism of action (Class III) (Álvarez-Sieiro et al., 2016). The enzymatic modification of Class I bacteriocins occur post-translationally and provide the molecules with uncommon amino acids and structures having an impact on their properties (e.g., lanthionine, heterocycles, head-to-tail cyclization, glycosylation) (Arnison et al., 2013). Class I bacteriocins containing unusual amino acids, such as lanthionine and/or (methyl)lanthionine are known as lantibiotics (Class Ia) (Álvarez-Sieiro et al., 2016). According to their genetic organization, lantibiotics have traditionally been classified in different classes (Arnison et al., 2013; Dischinger et al., 2013) and more recently in types (Álvarez-Sieiro et al., 2016).

*Lactobacillus plantarum* strains isolated from different environments have been reported to produce both lantibiotic and/or non-lantibiotic bacteriocins (González et al., 1994; Ruiz-Barba et al., 1994; Rekhif et al., 1995; Franz et al., 1998; Todorov and Dicks, 2005; Rojo-Bezares et al., 2007; Song et al., 2014). *L. plantarum* LL441, a dairy strain isolated among the dominant microbiota of a starter-free cheese made from raw milk, has been reported to produce a bacteriocin known as plantaricin C that inhibits several Gram-positive bacteria (González et al., 1994). Although first described as a, receptor-independent, pore-forming lantibiotic causing dissipation of the proton motive force and immediate release of pre-accumulated solutes in susceptible bacteria (González et al., 1996), plantaricin C has been shown to inhibit the cell wall biosynthesis by forming a complex with the peptidoglycan precursor lipid II (Wiedemann et al., 2006). The solution structure of plantaricin C has been shown to be a mature peptide of 27 amino acid residues, containing one dehydroalanine, one lanthionine and three β-methyl-lanthionine residues (Turner et al., 1999). However, the genetics of plantaricin C has remained elusive due to a lack of appropriate tools for use with *L. plantarum*. Fortunately, genome sequencing and analysis, which have become pivotal in deciphering the biochemical and technological potential of LAB strains (Langa et al., 2012; Cárdenas et al., 2015; Ziemert et al., 2016), now offers the possibility of investigating the genetic background of *L. plantarum* LL441 plantaricin C production, maturation, and secretion.

The present work reports an analysis of the draft genome sequence of *L. plantarum* LL441 and the genetic characterisation of the plantaricin C locus, which was located on a large plasmid, the molecular structure of which was also determined. The information presented could be of help in developing new biotechnological applications.

## Materials and Methods

### Strains and Culture Conditions

*Lactobacillus plantarum* LL441 and *Lactobacillus sakei* CECT906 were grown in MRS medium (Merck, Darmstadt, Germany) at 32°C under static aerobic conditions.

### Plantaricin C Production

Production of plantaricin C by *L. plantarum* LL441 was confirmed after growth of the strain in MRS for 24 h. Cells were removed by centrifugation and the supernatant brought to near-neutrality (pH = 6.5) with 0.1 N NaOH. It was then filter-sterilized through a 0.2 μm membrane (Merck). To quantify the antimicrobial activity of the plantaricin C, a dilution series (two-fold) of the supernatant was prepared for agar well diffusion tests against the susceptible control strain *L. sakei* CECT 906. The titre was defined as the reciprocal of the highest dilution showing definite inhibition of growth of the indicator lawn (expressed in arbitrary activity units; AAU).

To test resistance of the bacteriocin to high temperature, filter-sterilized, pH-neutralized supernatants were boiled for 15 min and assayed for antimicrobial activity as above. The susceptibility of plantaricin C to proteases was checked by incubating 50 μl of the supernatant with pronase, trypsin, or proteinase K (all from Sigma-Aldrich, St. Louis, MO, United States) at concentrations of 0.15–5 mg mL^-1^. Incubation proceeded for 30 min at 37°C for pronase and trypsin, and at 55°C for proteinase K. Protease-treated supernatants were assayed for activity as indicated above.

### Isolation of Genomic and Plasmid DNA

Total genomic DNA was extracted and purified from *L. plantarum* LL441 using the GenElute^TM^ Bacterial Genomic DNA kit (Sigma-Aldrich) following the manufacturer’s instructions for Gram-positive bacteria. The concentration and quality of the DNA was measured using an Epoch spectrophotometer (BioTek, Winooski, VT, United States).

The isolation of plasmid DNA from *L. plantarum* LL441 was performed according to the method of O’Sullivan and Klaenhammer (1993) with minor modifications (the denaturation and neutralization steps were performed using the solutions provided with the commercial Plasmid Mini Kit (Qiagen, Hilden, Germany). Plasmid profiles were observed by electrophoresis in 0.75% agarose gels in 1× TAE buffer (40 mM Tris, 20 mM acetic acid, and 1 mM EDTA), stained with ethidium bromide (0.5 mg mL^-1^), and visualized and photographed under UV light.

### Genome Sequencing, Annotation, and Analysis

A genomic library of 0.5 kbp was constructed from LL441 DNA and paired-end sequenced using a HiSeq 1000 System sequencer (Illumina, Inc., San Diego, CA, United States). Quality-filtered reads were assembled in contigs using Velvet software v.1.2.10 ^1^. The genome was annotated using the RAST annotation system^2^ and the NCBI Prokaryotic Genome Annotation Pipeline^3^. DNA and deduced protein sequences of interest were examined individually for homology against non-redundant DNA and protein databases using the on-line BLAST program^4^. The homology of deduced proteins was further investigated by searching the KEGG^5^, Uniprot^6^, and COG^7^ databases. Genomic assembled data were submitted to the GenBank database, under the accession no. LWKN00000000.1.

To identify and visualize plasmid-associated sequences, assembled contigs were analyzed using PLACNET (Vielva et al., 2017), a graph-based software tool for the reconstruction of plasmids from next generation sequence pair-end datasets via the creation of a network of contig interactions. Sequence alignments were performed by using Clustal X 2.0 (Larkin et al., 2007). Finally, web-based tools, such as BAGEL 4 (van Heel et al., 2018) and antiSMASH (Blin et al., 2017), dedicated to search for bacteriocin and secondary metabolite gene clusters, respectively, were used for the detection of open reading frames (ORFs) involved in the production of antimicrobial compounds.

### Hybridisation Experiments

Southern blot analysis was performed as described by Sambrook and Russell (2001). Briefly, total genomic DNA was extracted and purified from *L. plantarum* LL441, and then digested with PstI, EcoRI, or HindIII (Fermentas GmbH, Sankt Leon-Rot, Germany). After transferring to a Hybond nylon membrane (Amersham; GE Healthcare, Pittsburgh, PA., United States), the DNA was hybridized with a probe based on an internal segment of the coding sequence of the *lanM* gene amplified by PCR. The DIG System (a digoxigenin, non-radioactive nucleic acid labeling and detection system) (Roche, Mannheim, Germany) was used for probe labeling, according to the manufacturer’s instructions.

### Assembling the pLL441-1 Sequence

Putative plasmid sequences from *L. plantarum* LL441, as identified by PLACNET analysis, were retrieved from the whole genome sequence data. Primers based on sequences at the extremes of the contigs were designed and used in PCR reactions for sequence verification and gap closing, employing plasmid DNA from *L. plantarum* LL441 as a template. Amplifications were performed in 50 μl reaction mixtures containing 2 μl of purified DNA (≈50 ng), 25 μl of 2× Taq master Mix (Ampliqon, Odense, Denmark), 1.5 μl of each primer (10 μM), and 20 μl H_2_O. The PCR conditions were as follow: an initial denaturation cycle at 95°C for 5 min, 35 cycles of a denaturation step at 94°C for 30 s, an annealing step at 50°C for 1 min, an extension step at 72°C for 2 min, and a final extension cycle at 72°C for 10 min. PCR amplicons were examined in 1% agarose gels, and stained and photographed as above. Amplicons were purified using a commercial kit (GenElute PCR Clean-Up Kit; Sigma-Aldrich) and sequenced by cycle extension in an ABI 373 DNA sequencer (Applied Biosystems; Thermo Scientific, Waltham, MA, United States). Plasmids sequences were finally assembled using the Vector NTI computer program (Invitrogen; Thermo Scientific). Predicted new ORFs were then manually inspected for homology against the NCBI non-redundant DNA and protein databases using BLAST.

## Results

As expected, *L. plantarum* LL441 was shown to produce a temperature-resistant bacteriocin, susceptible to pronase and partially resistant to trypsin and proteinase K (data not shown). After 24 h of growth, plantaricin C titres of about 550 AAU were recorded, which were similar to those reported by González et al. (1994). After confirmation of plantaricin C production, total genomic DNA was isolated from *L. plantarum* LL441, purified, quantified, and subjected to genome sequencing.

### General Overview of the *L. plantarum* LL441 Genome

**Supplementary Table 1** summarizes the general features of the *L. plantarum* LL441 genome. Its sequencing and assembly produced 170 contigs with sizes ranging between 201 and 116,756 bp. The number of base pairs was 3,124,603 bp, and the GC content 44.52%. The RAST server identified 3,004 ORFs distributed in 337 subsystems, while the NCBI pipeline identified 3,017 genes, of which 2,935 were predicted to be coding sequences. All the 23S rRNA gene sequences were identical, while nucleotide heterogeneity was observed for genes encoding the 5S (at one position) and 16S (at two positions) molecules. Seventy-two tRNA coding sequences were found, corresponding to all 20 natural amino acids: Leu (7 sequences), Arg (6), Gly and Lys (5), Asn, Asp, Gln, Met, Pro, Ser, and Thr (4), Glu, His, Tyr, and Val (3), Ala, Ile, Phe, and Trp (2), and Cys (1). The presence of several phage-related ORFs in the examined genome, among which a likely complete prophage was identified (locus tags from A6B36_13490 to A6B36_13740), was noteworthy. This phage region showed strong homology to sequences in other *L. plantarum* genomes, especially those of *L. plantarum* C410L (CP017954.1) and *L. plantarum* X7021 (CP025412.1).

The potential link between the dairy origin and gene content of LL441 was analyzed by comparing the presence/absence of orthologous genes among the pangenome of 160 *L. plantarum* strains whose annotated genomes are available in databases. From the dendrogram generated (**Supplementary Figure 1**), grouping of the strains by origin was not observed. The pangenome of *L. plantarum* consisted in 16,672 genes, of which 841 could form the core genome (present in 99–100% of the strains). Gene content revealed LL441 to be quite similar to the human isolate *L. plantarum* HFC8 (BioProject PRJNA295007). As compared to all other strains, *L. plantarum* LL441 contained 63 strain-specific, unique genes. These were shown mostly to be composed by genes encoding total and partial integrases and recombinases, pseudogenes, and ORFs encoding hypothetical and phage-associated proteins (**Supplementary Table 2**).

**FIGURE 1:**
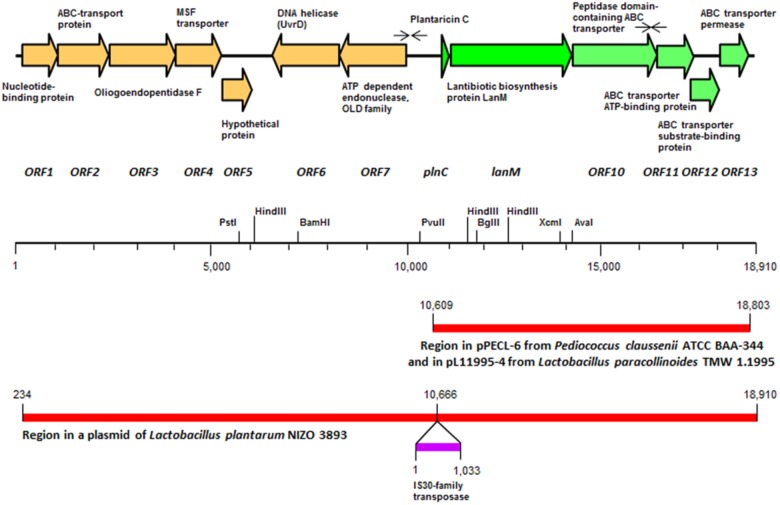
Schematic representation of the plantaricin C locus in *Lactobacillus plantarum* LL441, in which the orientation and size of the different open reading frames (ORFs) and the proteins they encode are indicated. Faced arrows above the ORFs indicate the presence of inverted repeat sequences resembling ρ-independent terminators. The position of relevant restriction sites in the contig and the segments of DNA homologous to those found in plasmids pPECL-6 from *Pediococcus claussenii* ATCC BAA-344 and pL11995-4 from *Lactobacillus paracollinoides* TMW 1.1995 and a plasmid from *L. plantarum* Nizo 3893 are also indicated.

### Characterisation of the Bacteriocin Locus

**FIGURE 2:**
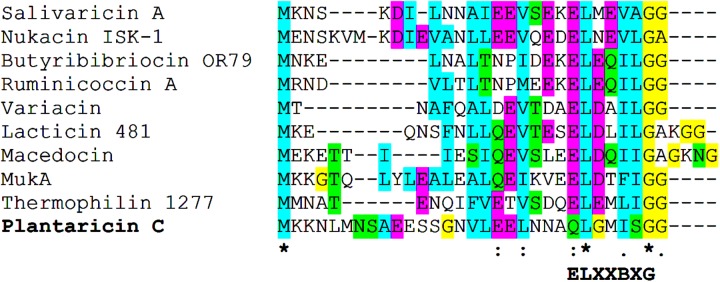
Sequence alignment of the precursor peptides of plantaricin C and those from selected Class Ia type II lantibiotics. Below the alignment, conserved amino acid positions (^∗^) and the ELXXBXG motif are indicated.

**FIGURE 3:**
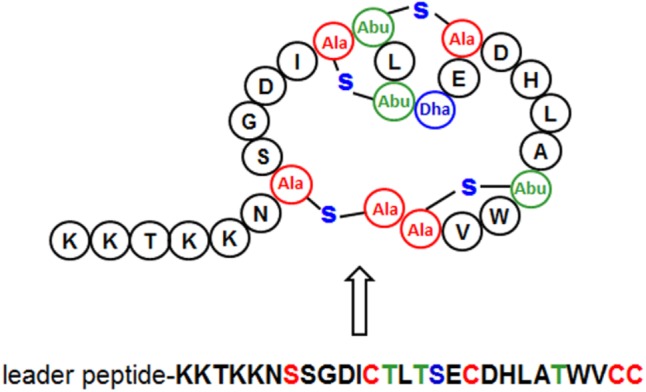
Primary (down) and secondary (up) structures of plantaricin C. Lanthionine (Ala), methyllanthionine (Abu), and Dehydroalanine (Dha) residues are color-coded and indicated. (Modified from Turner et al., 1999).

Scanning the entire draft genome of *L. plantarum* LL441 for a translated DNA sequence matching the amino acid sequence of plantaricin C revealed the product of a small ORF (locus tag A6B36_00026) located in a contig of 18,910 bp. The gene, named *plnC*, was found to be the first of a cluster of genes organized in an operon-like structure. **Figure 1** shows the genetic organization of this contig (locus tags A6B36_00005 through A6B36_00070 in GenBank accession no. LWKN00000000). The plantaricin C operon (in green in **Figure 1**) appeared to be formed by six ORFs (locus tags A6B36_00026 to A6B36_00005). The first ORF corresponded to the plantaricin C structural gene (*plnC*), with 174 nucleotides predicting a 58-amino acid precursor peptide composed of an N-terminal 31-amino acid leader peptide (MKKNLMNSAEESSGNVLEELNNAQLGMISGG) followed by a 27-amino acid core peptide (KKTKKNSSGDICTLTSECDHLATWVCC). The leader peptide, which may drive the secretion of plantaricin C, was seen to characteristically end in the double glycine (GG) motif reported for many bacteriocins and other exported cell signaling proteins produced by Gram-positive bacteria (Dirix et al., 2004). The leader peptide showed no significant sequence homology to similar regions of other proteins or bacteriocins (except for an equivalent peptide in plasmids from ATCC BAA-344, TMW 1.1995 and Nizo 3893 strains, see below), suggesting it to be highly specific for plantaricin C. Nonetheless, sequence alignment of the plantaricin C leader peptide with others from bacteriocins revealed partial structural homology to those of type II lantibiotics (Oman and van der Donk, 2010; Álvarez-Sieiro et al., 2016) and identified a sequence resembling the characteristic signal peptide ELXXBXG motif (-QLGMISG- in plantaricin C) (**Figure 2**). Maturation and secretion of the core peptide would result in the active form of the bacteriocin, a 27-amino acid residue containing several modified amino acids (Turner et al., 1999). The previously unidentified amino acids at positions 7, 12, 13, 15, 18, 23, 26, and 27 derived from the solution structure of plantaricin C (Turner et al., 1999) were found to be (on the basis of their encoding codons) Ser, Cys, Thr, Thr, Ser, Thr, Cys, and Cys, respectively. Based on these results, **Figure 3** depicts an amended version of sequence and structure of the plantaricin C as reported by Turner et al. (1999). Downstream of *plnC* was a long ORF (A6B36_00025), the derived amino acid sequence of which showed extensive homology to LanM proteins; these are thought to be involved in the maturation of type II lantibiotics. Downstream of *lanM*, four ORFs encoding components of ABC transporters were found (**Figure 1**), among which the first one contained a characteristic peptidase domain. The three downstream genes could encode for ABC proteins with ATP-binding, substrate-binding, and permease activity, respectively.

The whole region of the plantaricin C operon (8,195 bp) shared 91% nucleotide identity with a segment of the plasmid pPECL-6 from the brewery strain *Pediococcus claussenii* ATCC BAA-344. The partial sequence of pPECL-6 (GenBank Accession no. CP003143.1) was released as part of the genome sequencing project of its host strain (Accession no. NC_017017; Pittet et al., 2012). The same region is also present in plasmid pL11995-4 from *Lactobacillus paracollinoides* TMW 1.1995 (CP014928), a strain which, like *P. claussenii* ATCC BAA-244, was isolated from a brewery environment. Finally, the whole sequence of the plantaricin C contig was found to be present in a plasmid-derived contig from the genome sequencing project of *L. plantarum* Nizo 3893 (LUXJ010054.1). The homology was interrupted upstream of the 5′ end of the bacteriocin operon by an insertion sequence (IS) element of 1,033 bp, encoding a transposase of the IS30 family (**Figure 1**).

Alignment of all ORFs of the plantaricin C operon in *L. plantarum* LL441 by their start codons (SC) (**Supplementary Figure 2**) allowed the identification of putative gene translation signals (ribosome binding sites; RBSs). However, no canonical -35 (TTGACA) and -10 (TATAAT) promoter sequences separated by 17–21 nt were observed. Sequence analysis of the plantaricin C operon also identified two rho-independent terminator-like structures (facing arrowheads in **Figure 1**), consisting, respectively, of a 13 bp-long inverted repeat (IR) separated by a six nucleotides-long loop (AACGGCCATTAGG——CCTAATGGCCGTT; -16.56G ΔG kcal/mol) and a 10 bp-long adjacent IR (CTGGGCTTGATCAAGCCCAG; -15.70 ΔG kcal/mol). These putative terminators were found around 600 bp upstream of the SC of *plnC*, and 150 nt downstream of the SC of the gene encoding the ABC-transporter ATP-binding protein (ORF11 in **Figure 1**).

Genome analysis by dedicated software tools, such as BAGEL 4 and antiSMASH, did not identify in the LL441 genome any further putative bacteriocin gene cluster or genes involved in the synthesis of secondary metabolites with antimicrobial potential.

### Location of the Plantaricin C Locus in pLL441-1

To confirm previous hints for a plasmid location of the plantaricin C locus (Delgado and Mayo, 2003), the genome sequence data of *L. plantarum* LL441 was analyzed using PLACNET software, and DNA hybridisation experiments performed.

**FIGURE 4:**
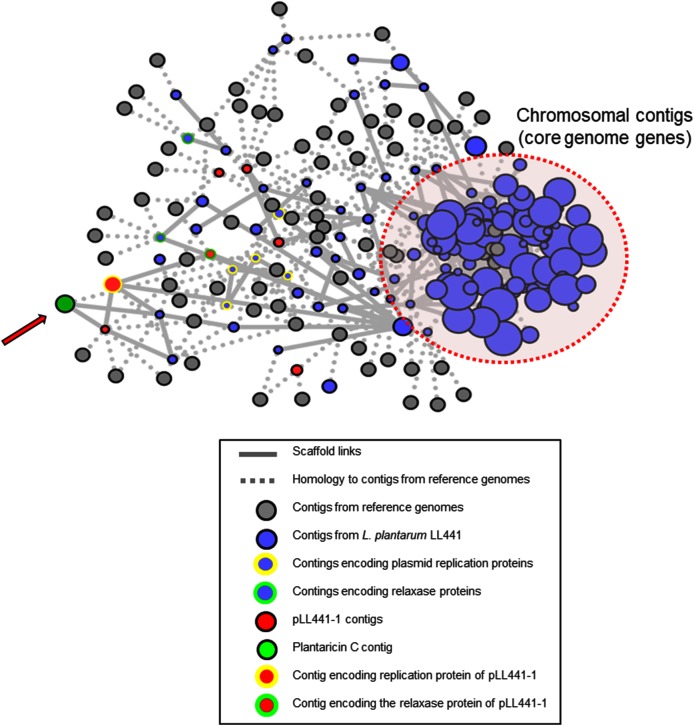
PLACNET reconstruction of the *L. plantarum* LL441 genome. Contigs are represented by blue nodes, while gray nodes represent reference genomes. The sizes of contig nodes are proportional to the contig length, while those of reference nodes are fixed. The doted red oval embraces contigs containing genes of the core genome. Colored node outlines represent contigs containing plasmid-specific protein genes (yellow, plasmid replication proteins; green, relaxase proteins). The arrow points toward the node of the contig harboring the plantaricin C operon.

Assembled contigs were first analyzed with PLACNET. This program returns results based on assembly information (including scaffold links and coverage), comparisons of sequences to known reference sequences, and plasmid diagnostic sequence features (Vielva et al., 2017). The program produces graphs with two types of nodes (assembled contigs and reference genomes) and two types of edges (scaffold links and homology to references). Reconstruction of the *L. plantarum* LL441 genome with PLACNET produced the diagram shown in **Figure 4**. The contig encoding plantaricin C (green in **Figure 4**) was arranged far away from contigs encoding chromosomal genes, and had interactions (solid bars) with contigs harboring plasmid-specific genes, strongly suggesting a plasmid location.

**FIGURE 5:**
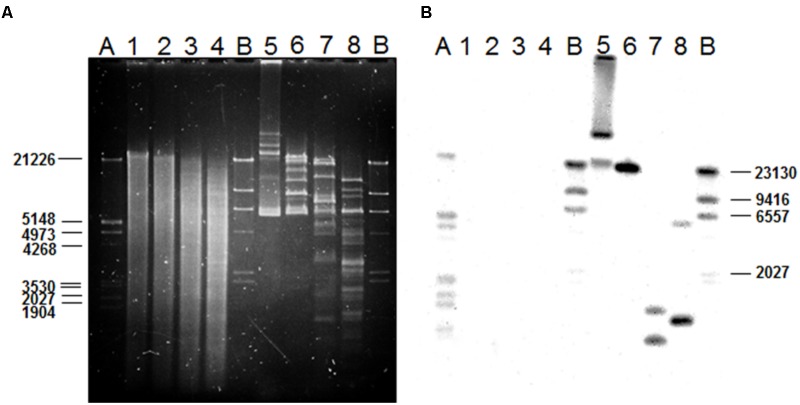
Gel electrophoresis of chromosomal and plasmid DNA from *L. plantarum* LL441 **(A)** and Southern blot results **(B)** of the gel in A after transferring of the DNA to a membrane and hybridizing with a digoxigenin-labeled probe (Roche) based on an internal 1-kbp fragment of the *lanM* gene (**Figure 1**) amplified by PCR. Order of the samples: 1, undigested chromosomal DNA from LL441; 2, 3, and 4, chromosomal DNA digested with PstI, EcoRI, and HindIII, respectively; 5, undigested plasmid DNA from LL441; 6, 7, and 8, plasmid DNA digested with PstI, EcoRI, and HindIII, respectively. A and B, pre-hybridized molecular weight markers: lambda DNA digested with PstI and lambda DNA digested with HindIII, respectively.

The PLACNET results, plus a search of the *L. plantarum* LL441 genome for genes encoding DNA replication initiation proteins, identified six ORFs encoding plasmid replication proteins belonging to different families (**Supplementary Figure 3**). The plasmid content of the bacterium (Delgado and Mayo, 2003), which showed six to eight plasmid bands reinforced these results. Total and plasmid DNA from *L. plantarum* LL441 was then subjected to hybridisation using as a probe an internal segment of *lanM* amplified by PCR and labeled with digoxigenin. Under the assay conditions, no hybridisation signal was obtained for chromosomal DNA, while a strong signal was seen when plasmid DNA was hybridized (**Figure 5**). In the undigested plasmid DNA sample (Line 5 in **Figure 5**), the strongest hybridisation signal appeared in a position corresponding to the largest plasmid of *L. plantarum* LL441. Hybridisation signals in undigested DNA are often found at the well level (plasmid molecules bound to undissolved material) and around the position of the chromosomal DNA (plasmids molecules broken during extraction). In the plasmid DNA sample digested with PstI, these multiple signals resolved into a single hybridisation band of 23–25 kbp (**Figure 5**, Line 6) -for which a single PstI fragment embraced the plantaricin C cluster (**Figure 1**). With both EcoRI and HindIII they resolved into two bands (**Figure 5**, Lines 7 and 8, respectively). The latter signals are those that would be expected from the results of contig sequence analysis.

**FIGURE 6:**
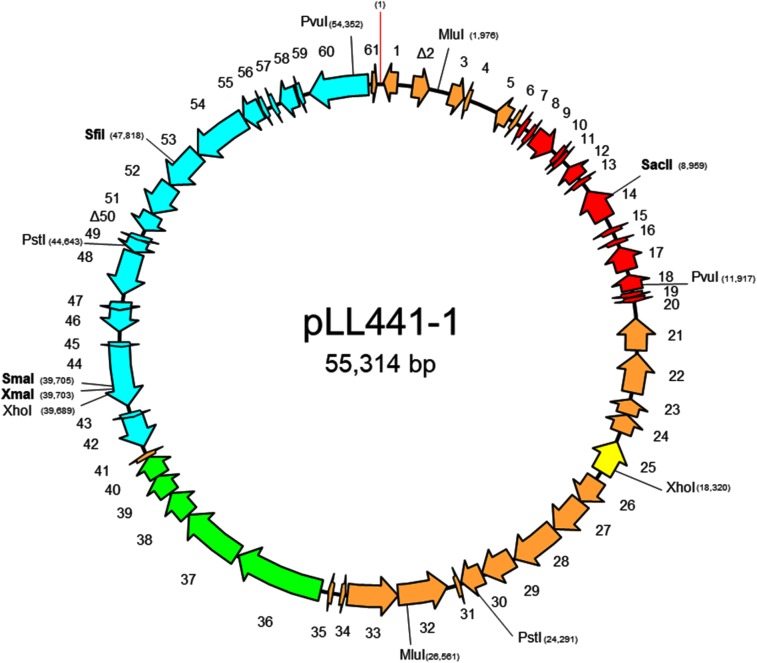
Genetic organization of plasmid pLL441-1, including position of relevant restriction enzymes and direction and length of genes and ORFs. Key of colors: in red, genes coding for proteins involved in plasmid replication, stability, and segregation; in yellow, ORFs of insertion sequences and integrase-related genes; in green, component genes of the plantaricin C locus; in pale blue, genes involved in conjugation; in pale brown, ORFs for other genes. ORFs overlapping to another by more than 20 amino acids or shorter than 50 amino acids in length were not taken into account.

Since the results strongly supported the idea that the genes involved in the plantaricin C synthesis, maturation and secretion are clustered in a region of the largest plasmid of *L. plantarum* LL441, primers based on sequences at the extremes of contigs assumed to be of plasmid origin were designed for use in PCR reactions. Amplicons were sequenced and the sequences assembled. As a result, a single circular sequence was obtained for the largest plasmid of *L. plantarum* LL441 (called pLL441-1; 55,314 bp). **Figure 6** shows its genetic organization, while **Supplementary Table 3** shows the ORF analysis for the whole molecule. The plasmid seems to be organized into four functional modules involved in (i) plasmid replication and control (in red in **Figure 6**), (ii) bacteriocin synthesis and processing (in green), (iii) conjugation (in pale blue), (iv) and other undefined functions (in pale brown). Comparison of pLL441-1 with plasmid-derived sequence from *L. plantarum* Nizo 3893 showed both plasmids sharing a region of about 34.5 kbp long (from ORF26 to ORF60 in pLL441-1; **Figure 6**) with high nucleotide identity (98–100%), while other segments of the two plasmids, including the replication region, showed lower (nucleotide identity <50%) or no homology.

## Discussion

The use of bacteriocins produced by LAB species as therapeutic agents or food preservatives (Dischinger et al., 2013; Chikindas et al., 2018) might be expected safe; most LAB enjoy “generally recognized as safe” (GRAS) and “qualified presumption of safety” (QPS) status and the structure of bacteriocins renders them susceptible to proteases (Álvarez-Sieiro et al., 2016). However, their biotechnological use demands we be in possession of thorough knowledge regarding their synthesis and regulation pathways, their biochemical properties, mode of action, and target organisms. The coding genes involved in their production, processing, immunity, and secretion also need to be characterized.

This study reports the draft genome sequence of *L. plantarum* LL441 and the genetic characterisation of the plantaricin C locus located on a 55-kbp plasmid (which was also assembled). With respect to gene content, the present results show the genome of *L. plantarum* LL441 to be closely related to the probiotic candidate *L. plantarum* HFC8 isolated in China from human feces (Kumari et al., 2015). As reported by other authors (Martino et al., 2016), *L. plantarum* strains of the same origin did not cluster together in the constructed dendrogram. Instead, those isolated from the same environment were found to be spread across the different branches, suggesting that gene distribution poorly reflects the origin of the strains. *L. plantarum* has recently been proposed a “nomadic” species that retains a functional set of genes that allow it to thrive in many different environments (Martino et al., 2016). The evolutionary history of *L. plantarum* appears to be complex, suggesting that the presence of key genes rather than gene content is involved in niche adaptation.

Plantaricin C, a lantibiotic produced by *L. plantarum* LL441, was isolated and biochemically characterized in the 1990s (González et al., 1994, 1996). Its solution structure was also elucidated (Turner et al., 1999). However, the genetics of this bacteriocin remained elusive. Its large number of amino acids with high codon redundancy hampered the construction of suitable primers and genetic probes with which to begin investigating. Analysis of LL441 genome identified the structural gene of plantaricin C, *plnC*, to be encoded in a contig of nearly 19-kbp long. It was followed by a large gene encoding a characteristic enzyme, LanM, which is commonly associated to gene clusters of the lacticin 481 group lantibiotics (Class Ia type II), such as those of cinnamycin, macedocin, mersacidin, mutacin II, and salivaricin A (Dufour et al., 2007; Begley et al., 2009). Furthermore, the whole plantaricin C operon resembles those of type II lantibiotics (lacticin 481, mutacin II, nukacin ISK-1, streptococcin A-FF22, macedocin, etc.). Operons from all these bacteriocins are composed by at least six genes, which include the bacteriocin structural gene, a lanM-like gene, and four downstream genes coding for ABC transporter subunits. These four ABC transporters, therefore, are thought to be involved in processing, secretion, and immunity (Dufour et al., 2007; Oman and van der Donk, 2010). The N-terminal region of LanM is involved in the dehydration of serine and threonine residues; while the C-terminal end catalyses the formation of the lanthionine rings (Begley et al., 2009). Bifunctional LanM enzymes with dehydratase and cyclization activities, also known as lantipeptide synthetases (Zhang et al., 2012), are prevalent in proteobacteria but uncommon in LAB species. Indeed, beyond *in silico* genome analysis, this is the first report of such an enzyme in a *Lactobacillus* species (Zhang et al., 2012).

Post-translational modification and secretion of lantibiotics rely on the recognition of the leader peptide for the enzymes involved in these processes (Oman and van der Donk, 2010). A highly specific leader peptide for plantaricin C was found, which has only equivalents to those in the bacteriocin-like operons of *L. plantarum* Nizo 3893 and in pPECL-6 and pL11995-4. In contrast to leader peptides from lantibiotics such as nisin, ericin, or gallidermin (Class Ia type I bacteriocins) (Álvarez-Sieiro et al., 2016), the plantaricin C signal sequence is rich in glutamic acid and ends in a characteristic double glycine (GG) motif, typical features of signal peptides of single-peptide, Class Ia type II lantibiotics (Dufour et al., 2007; Oman and van der Donk, 2010). Activation of lantibiotics usually occurs during secretion, which in turn may be undertaken with the aid of the ABC-type transporters (Twomey et al., 2002). Immunity against lantibiotics is also provided by the action of ABC transporters (Alkhatib et al., 2012). In the plantaricin C locus, four genes encoding components of ABC transporters were identified; the first of which contained the typical peptidase domain that might remove the leader peptide (Dufour et al., 2007).

Due to the short reads obtained with the Illumina sequencing technology used, and the limitations of the Velvet assemblage software employed (Zerbino and Birney, 2008), the contiguity of DNA segments containing repeated sequences, such as those of IS-like elements (which are abundant in the chromosome and plasmids of LAB species; Kleerebezem et al., 2003; Siezen et al., 2005; Górecki et al., 2011; Crowley et al., 2013; Flórez and Mayo, 2015), and others cannot always be resolved. Therefore, to locate the plantaricin C cluster, an *in silico* analysis was performed; this was complemented by hybridisation experiments and conventional PCR amplification and sequencing. Together, these techniques placed the plantaricin C cluster on the largest plasmid of *L. plantarum* LL441 (pLL441-1). The results confirmed our previous association of bacteriocin production and plasmid content in LL441 (Delgado and Mayo, 2003). pLL441-1 was composed of four functional modules, each encompassing approximately one quarter of the plasmid’s entire structure. Composed of cassettes coding for distinct functions, the mosaic nature of LAB plasmids (Siezen et al., 2005; Górecki et al., 2011; Wegmann et al., 2012; Flórez and Mayo, 2015), including those of *L. plantarum* (van Kranenburg et al., 2005; Chen et al., 2012; Xi et al., 2013) is well recognized. However, only a single IS256-like transposase of the IS*1310* family (ORF25) was found in pLL441-1. In contrast, several nearly identical intergenic sequences were found in the gaps between the contigs resolved by PCR amplification and sequencing. These repeated sequences might have interfered with the Velvet software assemblage of the reads. BLAST analysis revealed plasmids of *P. claussenii* (NC_017017; Pittet et al., 2012) and *L. paracollinoides* (CP014928) to have a region of high nucleotide identity to the sequence encoding plantaricin C in *L. plantarum* LL441. Further, genome analysis revealed a part of the pLL441-1 plasmid, including the plantaricin C gene cluster, to be embedded in a plasmid from *L. plantarum* Nizo 3893. In the latter plasmid an IS element was shown to be inserted 200 bp upstream of the plantaricin structural gene. Whether any of these three strains produces a bacteriocin similar to plantaricin C is currently unknown. Of note are a couple of nucleotide substitutions in the *plnC* genes of *P. claussenii* and *L. paracollinoides*, which drives two amino acid changes at positions 7 and 38 in the precursor peptide sequence. In both pPECL-6 and pL11995-4, the bacteriocin locus appears to be flanked by IS-like elements, suggesting horizontal acquisition. This is not so apparent in *L. plantarum* LL441, as no IS-derived segments were found at the extremes of the contig, and only one IS element was identified in the whole of pLL441-1. In this work, efforts for assembling plasmid-associated sequences focused on those composing pLL441-1; thus, yet interesting, all other five molecules remain still cryptic.

## Conclusion

The locus coding for the lantibiotic plantaricin C in *L. plantarum* LL441 was found to be encoded on an 18 kbp contig containing six genes organized in an operon-like structure. These included the bacteriocin structural gene and five downstream genes that probably encode the machinery involved in plantaricin C maturation, immunity and export. The plantaricin C precursor was shown to be a 58-amino acid residue peptide comprising a 31-amino acid double-glycine leader sequence plus a 27-amino acid precursor peptide. Hybridisation experiments showed the plantaricin C gene cluster to be encoded on the largest plasmid harbored by *L. plantarum* LL441 (pLL441-1). Conventional PCR amplification followed by sequencing was used for completing the sequence of the pLL441-1 molecule, which was shown to be composed of four functional modules of approximately equal length (each one quarter of the entire plasmid). Knowing the genetic structure and the coding region of plantaricin C will be of help in the molecular study of its components and their functions in the synthesis, modification, transport, and immunity. Further, it will be of use in the transfer of the genetic elements required for its synthesis to starter and probiotic LAB species and strains. These, or the purified lantibiotic itself, could be employed in new biotechnological and clinical applications.

## Author Contributions

BM and ABF conceived and designed the experiments, performed the experiments, analyzed the data, wrote, reviewed, and approved the final version of the manuscript. BM contributed to reagents, materials, and funding.

## Conflict of Interest Statement

The authors declare that the research was conducted in the absence of any commercial or financial relationships that could be construed as a potential conflict of interest.

## Footnotes

1 https://www.ebi.ac.uk/∼zerbino/velvet/

2 http://rast.nmpdr.org/

3 http://www.ncbi.nlm.nih.gov/genome/annotation_prok/

4 http://blast.ncbi.nlm.nih.gov/Blast.cgi

5 http://www.genome.jp/kegg/pathway.html

6 http://www.uniprot.org

7 http://www.ncbi.nlm.nih.gov/COG
