# Supplementary material for: Genome Analysis of Lactobacillus plantarum LL441 and Genetic Characterisation of the Locus for the Lantibiotic Plantaricin C
Source: Front Microbiol. 2018 Aug 17;9:1916. doi: 10.3389/fmicb.2018.01916 (PMC6107846; doi:10.3389/fmicb.2018.01916)
Supplement: Supplementary file 1 [file Table_1.docx]

**Supplementary Table 1.-** General features of the *Lactobacillus plantarum* LL441 genome

| **Feature/gene(s) coding for** | **Figures** |
| --- | --- |
|  |  |
| Size (bp) | 3,124,603 |
| GC content (%) | 44.52 |
| No. of contigs | 170 |
|  |  |
| No. of total genes (NCBI)^a^ | 3,017 |
| No. of total coding sequences (NCBI) | 2,935 |
| No. of coding genes (NCBI) | 2,708 |
| No. of pseudogenes (NCBI) | 227 |
| No. subsystems (RAST)^b^ | 337 |
| No. of unique genes | 63 |
| rRNA | 6 (2x5S, 3x16S, 1x23S) |
| tRNA | 72 |
| non-coding RNA (ncRNA) | 4 |
|  |  |
| ABC superfamily transporter | 231 |
| Proteins involved in carbohydrate utilization and transport | 215 |
| Monosaccharide transport/utilization | 26 |
| Di- and oligo-saccharides transport/utilization | 63 |
| Polysaccharides utilization | 6 |
| β-galactosidase | 4 (one with L and M peptides) |
| 6-phospho-β-galactosidase | 1 |
| β-glucosidase | 3 |
| 6-phospho-β-glucosidase | 5 |
| α-galactosidase | 4 |
| α-glucosidase | 4 |
| α-rhamnosidase | 2 |
| α-amylase | 2 |
| Proteases/proteinases/hydrolases | 74 |
| Aminopeptidase | 12 |
| Carboxypeptidase | 9 |
| Di- tri-peptidase | 4 |
| Extracellular zinc metalloproteinase | 4 |
| Transposase/resolvase/integrase/nickase proteins | 79 |
| Phage-related proteins | 69 (39 from a complete prophage) |
| Heavy metal (Cu, Co, As, Hg) homeostasis proteins | 17 |
| Competence-associated proteins | 11 |
| Conjugation-related proteins | 12 (possibly 19; one operon) |
| Capsular exopolysaccharide family protein | 10 (one operon) |
| Plasmid initiation replication proteins | 6 |
| Proteins for antibiotic resistance | 0 |
|  |  |

^a^NCBI, NCBI Prokaryotic Genome Annotation Pipeline.

^b^RAST, Rapid Annotation using Subsystem Technology Server.
