# Supplementary material for: Genome Analysis of Lactobacillus plantarum LL441 and Genetic Characterisation of the Locus for the Lantibiotic Plantaricin C
Source: Front Microbiol. 2018 Aug 17;9:1916. doi: 10.3389/fmicb.2018.01916 (PMC6107846; doi:10.3389/fmicb.2018.01916)
Supplement: Supplementary file 2 [file Table_2.DOCX]

| **Supplementary Table 2.-** Sixty-three unique ORFs in the *Lactobacillus plantarum* LL441 genome and comparison to their deduced proteins to other in databases. | | | | | | | | | |
| --- | --- | --- | --- | --- | --- | --- | --- | --- | --- |
| **Locus tag** | **Strand** | **Start** | **End^a^** | **Length (aa)^b^** | **Contig accession no.** | **Protein name** | **Protein identity** | **Identities (%)** | **PLACNET location** |
|  |  |  |  |  |  |  |  |  |  |
| A6B36_00030 | + | 8350 | 8655 | 101 | NZ_LWKN01000001 | Hypothetical protein | WP_052661614 | 101/101 (100%) | pLL441-1 |
| A6B36_01170 | + | 1116 | 1754 | 212 | NZ_LWKN01000009 | Hypothetical protein | WP_070084825.1 | 212/212 (100%) | pLL441-1 |
| A6B36_01175 | + | 1763 | 2317 | 184 | NZ_LWKN01000009 | Recombinase family protein | WP_070084826.1 | 184/184 (100%) | pLL441-1 |
| A6B36_01180 | + | 2541 | 3896 | 451 | NZ_LWKN01000009 | NAD(P)/FAD-dependent oxidoreductase | WP_070084827.1 | 451/451 (100%) | pLL441-1 |
| A6B36_01185 | + | 3978 | 5105 | 375 | NZ_LWKN01000009 | Hypothetical protein | WP_070084828.1 | 375/375 (100%) | pLL441-1 |
| A6B36_01740 | + | 1004 | 1529 | 161 | NZ_LWKN01000017 | DUF536 domain-containing protein | WP_070084862.1 | 78/79 (99%) | Plasmid |
| A6B36_01745 | + | 1845 | 3581 | 578 | NZ_LWKN01000017 | Hypothetical protein | WP_070084863.1 | 578/578 (100%) | Plasmid |
| A6B36_02160 | + | 533 | 1912 | 459 | NZ_LWKN01000020 | Pyridine nucleotide-disulfide oxidoreductase | WP_070084880.1 | 459/459 (100%) | Chromosome |
| A6B36_02385 | + | 166 | 1965 | 599 | NZ_LWKN01000024 | Cadmium-translocating P-type ATPase | WP_070084890.1 | 599/599 (100%) | Plasmid |
| A6B36_02415 | - | 6785 | 7381 | 198 | NZ_LWKN01000024 | Recombinase family protein | WP_003582117.1 | 198/198 (100%) | Plasmid |
| A6B36_02420 | + | 7560 | 10553 | - | NZ_LWKN01000024 | Pseudogene | - | - | Plasmid |
| A6B36_02445 | + | 13472 | 14259 | - | NZ_LWKN01000024 | Pseudogene | - | - | Plasmid |
| A6B36_02450 | - | 14404 | 15582 | 392 | NZ_LWKN01000024 | Phosphoribosyl-aminoimidazole carboxamide formyltransferase | WP_070084893.1 | 392/392 (100%) | Plasmid |
| A6B36_02455 | + | 15941 | 16183 | 80 | NZ_LWKN01000024 | Hypothetical protein | WP_057905708.1 | 80/80 (100%) | Plasmid |
| A6B36_02460 | + | 16205 | 16459 | - | NZ_LWKN01000024 | Pseudogene | - | - | Plasmid |
| A6B36_03150 | - | 13620 | 14234 | 204 | NZ_LWKN01000035 | Hypothetical protein | WP_070084932.1 | 204/204 (100%) | pLL441-1 |
| A6B36_03350 | - | 3002 | 3880 | 292 | NZ_LWKN01000041 | DUF3037 domain-containing protein | WP_070084940.1 | 292/292 (100%) | Chromosome |
| A6B36_03355 | - | 3844 | 4869 | 341 | NZ_LWKN01000041 | Hypothetical protein | WP_070084941.1 | 341/341 (100%) | Chromosome |
| A6B36_03885 | + | 1357 | 1833 | 158 | NZ_LWKN01000046 | DUF536 domain-containing protein | WP_070084968.1 | 158/158 (100%) | pLL441-1 |
| A6B36_04215 | + | 1 | 306 | 101 | NZ_LWKN01000055 | Transposase, partial | EMP43495.1 | 101/123 (100%) | - |
| A6B36_04220 | + | 394 | 591 | 65 | NZ_LWKN01000055 | Hypothetical protein | AUI77366.1 | 49/111 (82%) | - |
| A6B36_04225 | - | 662 | 1141 | 159 | NZ_LWKN01000056 | Hypothetical protein | WP_068222442.1 | 159/159(100%) | Plasmid |
| A6B36_04230 | + | 1509 | 1742 | - | NZ_LWKN01000056 | Pseudogene | - | - | Plasmid |
| A6B36_05215 | - | 97 | 1725 | 542 | NZ_LWKN01000065 | Hypothetical protein | WP_070085038.1 | 542/542 (100%) | Plasmid |
| A6B36_05225 | + | 99 | 1172 | 357 | NZ_LWKN01000066 | IS30 family transposase | WP_070085039.1 | 357/357 (100%) | Plasmid |
| A6B36_05335 | + | 843 | 1340 | 165 | NZ_LWKN01000068 | GNAT family N-acetyltransferase | WP_080481576.1 | 165/165 (100%) | Plasmid |
| A6B36_05345 | - | 2435 | 3649 | 404 | NZ_LWKN01000068 | Hypothetical protein | WP_080481575.1 | 404/404 (100%) | Plasmid |
| A6B36_05350 | + | 428 | 787 | 119 | NZ_LWKN01000069 | β-galactosidase GanA (*Lactobacillus parabuchneri*), partial | ORN06308.1 | 93/136 (99%) | Chromosome |
| A6B36_05355 | + | 1 | 306 | 101 | NZ_LWKN01000070 | IS5 family transposase, partial | WP_010014206.1 | 101/123 (100%) | - |
| A6B36_06210 | - | 150473 | 151066 | 197 | NZ_LWKN01000077 | GTP-binding protein | WP_003640795.1 | 197/197 (100%) | Chromosome |
| A6B36_06345 | + | 3900 | 4124 | 74 | NZ_LWKN01000084 | Hypothetical protein | WP_070085095.1 | 74/74 (100%) | pLL441-1 |
| A6B36_06350 | + | 4323 | 4799 | 158 | NZ_LWKN01000084 | GNAT family N-acetyltransferase | WP_070085096.1 | 158/158 (100%) | pLL441-1 |
| A6B36_06360 | - | 5128 | 5340 | 70 | NZ_LWKN01000084 | Hypothetical protein | WP_070085098.1 | 70/70 (100%) | pLL441-1 |
| A6B36_06945 | - | 66887 | 67701 | 271 | NZ_LWKN01000091 | Citrate lyase subunit beta, partial | WP_070085123.1 | - | Chromosome |
| A6B36_08310 | + | 50555 | 51007 | 150 | NZ_LWKN01000099 | Hypothetical protein | WP_070085187.1 | 150/150 (100%) | Chromosome |
| A6B36_09545 | + | 455 | 760 | 101 | NZ_LWKN01000104 | Hypothetical protein “prophage” | WP_070085263.1 | 101/101 (100%) | Chromosome |
| A6B36_09550 | + | 750 | 1040 | 96 | NZ_LWKN01000104 | Hypothetical protein | WP_070085264.1 | 96/96 (100%) | Chromosome |
| A6B36_09555 | + | 1041 | 1718 | - | NZ_LWKN01000104 | Pseudogene | - | - | - |
| A6B36_09560 | + | 3818 | 4972 | 384 | NZ_LWKN01000104 | LysM peptidoglycan-binding domain-containing protein | WP_070085283.1 | 384/384 (100%) | Chromosome |
| A6B36_09570 | + | 5256 | 5633 | 125 | NZ_LWKN01000104 | Holin | WP_070085267.1 | 125/125 (100%) | Chromosome |
| A6B36_09575 | - | 5853 | 6302 | 149 | NZ_LWKN01000104 | Hypothetical protein | WP_080481590.1 | 149/149 (100%) | Chromosome |
| A6B36_09580 | - | 6293 | 6670 | 125 | NZ_LWKN01000104 | Hypothetical protein | WP_070085268.1 | 125/125 (100%) | Chromosome |
| A6B36_09585 | - | 6682 | 7290 | 202 | NZ_LWKN01000104 | Hypothetical protein | WP_070085269.1 | 202/202 (100%) | Chromosome |
| A6B36_10190 | + | 27259 | 27648 | 129 | NZ_LWKN01000108 | MarR family transcriptional regulator | WP_003643287.1 | 129/129 (100%) | Chromosome |
| A6B36_10220 | + | 31 | 212 | 60 | NZ_LWKN01000115 | Transposase, partial | WP_003581649.1 | - | - |
| A6B36_10705 | - | 659 | 1597 | 312 | NZ_LWKN01000132 | Serine/threonine protein kinase, partial | WP_003643440.1 | - | Chromosome |
| A6B36_11835 | + | 1082 | 1477 | 131 | NZ_LWKN01000147 | MFS transporter | WP_070085371.1 | 131/131 (100%) | Chromosome |
| A6B36_12395 | + | 185 | 988 | 267 | NZ_LWKN01000150 | Peptide-methionine (S)-S-oxide reductase | WP_070085398.1 | 267/267 (100%) | Chromosome |
| A6B36_12770 | - | 16622 | 17047 | 141 | NZ_LWKN01000153 | Hypothetical protein | WP_070085422.1 | 141/141 (100%) | Chromosome |
| A6B36_13230 | - | 84877 | 85095 | - | NZ_LWKN01000156 | Pseudogene | - | - | Chromosome |
| A6B36_13490 | - | 147329 | 147703 | 124 | NZ_LWKN01000156 | Holin “phage” | WP_080481610.1 | 124/124 (100%) | Chromosome |
| A6B36_13510 | - | 150430 | 150690 | 86 | NZ_LWKN01000156 | Hypothetical protein | WP_070085463.1 | 86/86 (100%) | Chromosome |
| A6B36_13515 | - | 150683 | 150406 | 907 | NZ_LWKN01000156 | Hypothetical protein | WP_070085464.1 | 907/907 (100%) | Chromosome |
| A6B36_13525 | - | 155878 | 157584 | 568 | NZ_LWKN01000156 | Phage tail protein | WP_070085466.1 | 568/568 (100%) | Chromosome |
| A6B36_13530 | - | 157658 | 162550 | 1630 | NZ_LWKN01000156 | Peptidase M23 “phageMin_tail superfamily” | WP_070085467.1 | 1630/1630 (100%) | Chromosome |
| A6B36_13545 | - | 163262 | 163903 | 213 | NZ_LWKN01000156 | Phage tail protein | WP_070085469.1 | 213/213 (100%) | Chromosome |
| A6B36_13550 | - | 163918 | 164928 | 126 | NZ_LWKN01000156 | DUF806 family protein | WP_070085470.1 | 126/126 (100%) | Chromosome |
| A6B36_13615 | - | 173904 | 174404 | 166 | NZ_LWKN01000156 | Hypothetical protein | WP_070085499.1 | 166/166 (100%) | Chromosome |
| A6B36_13625 | - | 175043 | 175816 | 257 | NZ_LWKN01000156 | Site-specific DNA-methyltransferase | WP_080481608.1 | 257/257 (100%) | Chromosome |
| A6B36_13695 | + | 183335 | 184111 | 258 | NZ_LWKN01000156 | Hypothetical protein | WP_070085490.1 | 258/258 (100%) | Chromosome |
| A6B36_13720 | + | 185437 | 185808 | 123 | NZ_LWKN01000156 | Hypothetical protein | WP_070085492.1 | 123/123 (100%) | Chromosome |
| A6B36_13735 | - | 186581 | 187156 | 191 | NZ_LWKN01000156 | Hypothetical protein | WP_070085493.1 | 191/191 (100%) | Chromosome |
| A6B36_14820 | - | 38 | 316 | 92 | NZ_LWKN01000166 | Hypothetical protein | WP_014216278.1 | 92/92 (100%) | Plasmid |

ªWithout stop codon.

^b^aa, amino acids.
