# Supplementary material for: Genome Analysis of Lactobacillus plantarum LL441 and Genetic Characterisation of the Locus for the Lantibiotic Plantaricin C
Source: Front Microbiol. 2018 Aug 17;9:1916. doi: 10.3389/fmicb.2018.01916 (PMC6107846; doi:10.3389/fmicb.2018.01916)
Supplement: Supplementary file 3 [file Table_3.docx]

| **Supplementary Table 3.-** Open reading frames (ORFs) analysis of pLL441-1, the plasmid that encodes the lantibiotic bacteriocin plantaricin C in *Lactobacillus plantarum* LL441. | | | | | | | | | |
| --- | --- | --- | --- | --- | --- | --- | --- | --- | --- |
| **ORF** | **5’ start position** | **3’ end position^a^** | **% GC content** | **No. of aa^b^** | **Known protein with the highest homology (microorganism)** | **% aa identity (identity length/total length)** | **GenBank Accession no.** | **GenBank locus_tag LL441^c^** | **GenBank protein_id LL441^c^** |
|  |  |  |  |  |  |  |  |  |  |
| ORF1 | 583 | 95 | 34.0 | 163 | Hypothetical protein (*Lactobacillus plantarum*) | (99%) 162/163 | WP_015063552 | A6B36_05015 | OEZ35684 |
| ΔORF2 | 1083 | 1682 | 39.8 | 200 | Replication initiation protein (RepB) (*L. plantarum*) | (99%) 197/261 | WP_076661597 | A6B36_05020 | Pseudogene |
| ORF3 | 2400 | 2915 | 33.1 | 172 | Hypothetical protein (*L. plantarum*) | (100%) 172/172 | KZU17828 | A6B36_05025 | OEZ35685 |
| ORF4 | 3439 | 3319 | 37.8 | 125 | DUF1093 domain-containing protein (*L. plantarum*) | (100%) 125/125 | WP_100255592 | A6B36_05030 | OEZ35686 |
| ORF5 | 4620 | 4147 | 37.1 | 158 | DUF536 domain-containing protein (*L. plantarum*) | (100%) 158/158 | WP_070084968 | A6B36_03885 | OEZ35901 |
| ORF6 | 4859 | 5068 | 32.9 | 70 | DUF3923 domain-containing protein (*Lactobacillus*) | (100%) 70/70 | WP_015063544 | A6B36_03880 | OEZ35900 |
| ORF7 | 5536 | 5195 | 36.0 | 114 | Type II toxin-antitoxin system, PemK-MazF family toxin (Bacteria) | (100%) 114/114 | WP_001748110 | A6B36_03875 | OEZ35899 |
| ORF8 | 5793 | 5533 | 35.2 | 87 | PbsX family transcriptional regulator (Lactobacillaceae) | (100%) 87/87 | WP_003643337 | A6B36_03870 | OEZ35898 |
| ORF9 | 5672 | 6463 | 36.6 | 264 | Tyrosine recombinase (*Lactobacillus paucivorans*) | (99%) 263/264 | KRO04657 | - | - |
| ORF10 | 6734 | 6943 | 31.0 | 70 | Hypothetical protein (*L. plantarum*) | (100%) 70/70 | WP_070085098 | A6B36_06360 | OEZ35460 |
| ORF11 | 6951 | 7238 | 34.1 | 92 | Hypothetical protein (*L. plantarum*) | (100%) 92/92 | WP_070085097 | A6B36_06355 | OEZ35459 |
| ORF12 | 7751 | 7278 | 33.6 | 158 | N-Acetyltransferase (*L. plantarum*) | (100%) 158/158 | WP_070085096 | A6B36_06350 | OEZ35458 |
| ORF13 | 8174 | 7953 | 40.8 | 74 | Hypothetical protein (*L. plantarum*) | (100%) 74/74 | WP_070085095 | A6B36_06345 | OEZ35457 |
| ORF14 | 9422 | 8422 | 38.8 | 367 | Plasmid replication initiation protein (*L. plantarum*) | (100%) 367/367 | WP_070085094 | A6B36_06340 | OEZ35456 |
| ORF15 | 10308 | 9931 | 35.5 | 126 | Hypothetical protein (*L. plantarum*) | (100%) 126/126 | WP_070085093 | A6B36_06335 | OEZ35455 |
| ORF16 | 10570 | 10331 | 30.5 | 80 | Hypothetical protein (*Lactobacillus*) | (100%) 80/80 | WP_062688575 | A6B36_06330 | OEZ35454 |
| ORF17 | 11383 | 10577 | 36.8 | 269 | ParA family protein (*L. plantarum*) | (100%) 269/269 | WP_070085092 | A6B36_06325 | OEZ35453 |
| ORF18 | 12073 | 11543 | 41.6 | 177 | Site-specific integrase, partial (*L. plantarum*) | (100%) 177/177 | WP_070085091 | A6B36_06320 | OEZ35452 |
| ORF19 | 12203 | 12478 | 37.7 | 92 | Type II toxin-antitoxin system, Phd-YefM family antitoxin (*L. plantarum*) | (98%) 91/92 | WP_003586674 | A6B36_03065 | OEZ36050 |
| ORF20 | 12481 | 14177 | 37.0 | 118 | Type II toxin-antitoxin system, YafQ family toxin  (*L. plantarum*) | (100%) 118/118 | WP_070084829 | A6B36_01190 | OEZ36399 |
| ORF21 | 14177 | 13053 | 28.2 | 375 | Hypothetical protein (*L. plantarum*) | (100%) 375/375 | WP_070084828 | A6B36_01185 | OEZ36398 |
| ORF22 | 15614 | 14262 | 39.9 | 451 | NAD/FAD-dependent oxidoreductase (*L. plantarum*) | (99%) 450/451 | WP_070084827 | A6B36_01180 | OEZ36397 |
| ORF23 | 16392 | 15841 | 42.1 | 184 | Recombinase family protein (*L. plantarum*) | (100%) 184/184 | WP_070084826 | A6B36_01175 | OEZ36396 |
| ORF24 | 17039 | 16404 | 30.5 | 212 | Hypothetical protein (*L. plantarum*) | (99%) 211/212 | WP_070084825 | A6B36_01170 | OEZ36395 |
| ORF25 | 18556 | 17384 | 44.9 | 391 | IS*256*-like element, IS*1310* family transposase  (*L. plantarum*) | (98%) 385/391 | WP_085776406 | A6B36_01165 | OEZ36394 |
| ORF26 | 18855 | 19790 | 30.6 | 312 | ThiF family adenylyltransferase (*L. plantarum*) | (100%) 312/312 | WP_052661616 | A6B36_00070 | OEZ36623 |
| ORF27 | 19786 | 21081 | 28.8 | 432 | Hypothetical protein (*L. plantarum*) | (100%) 432/432 | WP_070084767 | A6B36_00065 | OEZ36622 |
| ORF28 | 21099 | 22796 | 29.8 | 566 | Hypothetical protein (*L. plantarum*) | (100%) 566/566 | WP_045352946 | A6B36_00060 | OEZ36621 |
| ORF29 | 22796 | 23959 | 29.2 | 388 | MFS transporter (*L. plantarum*) | (99%) 387/388 | WP_045352944 | A6B36_00055 | OEZ36620 |
| ORF30 | 23949 | 24704 | 31.4 | 252 | Class I SAM-dependent methyltransferase  (*L. plantarum*) | (100%) 252/252 | WP_045352942 | A6B36_00050 | OEZ36619 |
| ORF31 | 24961 | 24830 | 33.4 | 44 | DNA recombinase (*L. plantarum*) | (100%) 44/44 | WP_045352940 | A6B36_00045 | OEZ36618 |
| ORF32 | 26935 | 25208 | 34.5 | 576 | ATP-dependent helicase (*L. plantarum*) | (100%) 576/576 | WP_045352938 | A6B36_00040 | OEZ36617 |
| ORF33 | 28662 | 26935 | 30.4 | 576 | ATP-dependent endonuclease (*L. plantarum*) | (100%) 576/576 | WP_045352936 | A6B36_00035 | OEZ36616 |
| ORF34 | 29118 | 28816 | 31.3 | 101 | Hypothetical protein (*L. plantarum*) | (100%) 101/101 | WP_052661614 | A6B36_00030 | OEZ36615 |
| ORF35 | 29338 | 29511 | 34.0 | 58 | Plantaricin C family lantibiotic (*L. plantarum*) | (100%) 58/58 | WP_064511516 | A6B36_00026 | OEZ36624 |
| ORF36 | 29594 | 32683 | 31.5 | 1030 | Type 2 lantipeptide synthetase LanM (*L. plantarum*) | (100%) 1030/1030 | WP_045352933 | A6B36_00025 | OEZ36614 |
| ORF37 | 32724 | 34880 | 30.7 | 719 | Peptidase domain-containing ABC transporter  (*L. plantarum*) | (99%) 718/719 | WP_045352932 | A6B36_00020 | OEZ36613 |
| ORF38 | 34883 | 35824 | 35.3 | 314 | Bacitracin ABC transporter ATP-binding protein  (*L. plantarum*) | (100%) 314/314 | WP_045352929 | A6B36_00015 | OEZ36612 |
| ORF39 | 35820 | 36554 | 36.4 | 245 | ABC transporter permease (*L. plantarum*) | (100%) 245/245 | WP_045352927 | A6B36_00010 | OEZ36611 |
| ORF40 | 36560 | 37294 | 33.3 | 245 | ABC transporter permease (*L. plantarum*) | (100%) 245/245 | WP_045352925 | A6B36_00005 | OEZ36610 |
| ORF41 | 37387 | 37572 | 44.1 | 62 | Hypothetical protein (*Lactobacillus*) | (97%) 60/62 | WP_016370329 | - | - |
| ORF42 | 38798 | 37677 | 42.7 | 374 | Primase, LtrC-like protein (*L. plantarum*) | (100%) 374/374 | WP_063488785 | A6B36_03070 | - |
| ORF43 | 39017 | 38805 | 41.8 | 71 | Hypothetical protein (*L. plantarum*) | (100%) 71/71 | WP_063488786 | A6B36_03075 | OEZ36033 |
| ORF44 | 41274 | 39142 | 42.5 | 711 | Type IA DNA topoisomerase (*L. plantarum*) | (100%) 711/711 | WP_063488787 | A6B36_03080 | OEZ36034 |
| ORF45 | 41691 | 41284 | 40.5 | 136 | Hypothetical protein (*L. plantarum*) | (100%) 136/136 | WP_045352995 | A6B36_03085 | OEZ36035 |
| ORF46 | 42572 | 41709 | 41.4 | 285 | Conjugal transfer protein (*L. plantarum*) | (100%) 285/285 | WP_080335181 | A6B36_03090 | OEZ36049 |
| ORF47 | 42958 | 42572 | 33.6 | 124 | Hypothetical protein (*L. plantarum*) | (100%) 124/124 | WP_080481563 | A6B36_03095 | OEZ36036 |
| ORF48 | 44469 | 42961 | 42.3 | 503 | Conjugal transfer protein (*L. plantarum*) | (100%) 503/503 | WP_070084926 | A6B36_03100 | OEZ36037 |
| ORF49 | 44941 | 44474 | 41.9 | 156 | Protein TrsJ, conjugal transfer protein (*L. plantarum*) | (100%) 156/156 | WP_063489628 | A6B36_03105 | OEZ36038 |
| ΔOR50 | 45343 | 45110 | 41.0 | 78 | Hypothetical protein FD10_GL002635  (*L. plantarum*) | (100%) 78/133 | KRL98968 | A6B36_03110 | Pseudogene |
| ORF51 | 45914 | 45300 | 41.4 | 205 | Hypothetical protein (*L. plantarum*) | (100%) 205/205 | WP_070084927 | A6B36_03115 | OEZ36039 |
| ORF52 | 47083 | 45932 | 46.5 | 384 | CHAP domain-containing protein (*L. plantarum*) | (100%) 384/384 | WP_070084928 | A6B36_03120 | OEZ36040 |
| ORF53 | 48502 | 47087 | 43.7 | 472 | Conjugal transfer protein (*L. plantarum*) | (100%) 472/472 | WP_070084929 | A6B36_03125 | OEZ36041 |
| ORF54 | 50513 | 48498 | 41.9 | 672 | Helicase, DUF87 domain-containing protein  (*L. plantarum*) | (100%) 659/659 | WP_070084930 | A6B36_03130 | OEZ36042 |
| ORF55 | 51184 | 50528 | 41.3 | 219 | Protein TrsD conjugal transfer protein | (100%) 219/219 | WP_016381866 | A6B36_03135 | OEZ36043 |
| ORF56 | 51515 | 51156 | 40.9 | 120 | Protein TrsC conjugal transfer protein | (100%) 120/120 | WP_014216295 | A6B36_03140 | OEZ36044 |
| ORF57 | 51871 | 51539 | 46.8 | 111 | Conjugal transfer protein (*L. plantarum*) | (100%) 111/111 | WP_070084931 | A6B36_03145 | OEZ36045 |
| ORF58 | 52487 | 52487 | 41.6 | 204 | Hypothetical protein (*L. plantarum*) | (100%) 204/204 | WP_070084932 | A6B36_03150 | OEZ36046 |
| ORF59 | 52837 | 52526 | 42.9 | 104 | Hypothetical protein (*L. plantarum*) | (99%) 103/104 | WP_070084933 | A6B36_03155 | OEZ36047 |
| ORF60 | 54979 | 52922 | 38.9 | 686 | Nickase (*L. plantarum*) | (100%) 686/686 | WP_070084934 | A6B36_03160 | OEZ36048 |
| ORF61 | 55093 | 55242 | 37.3 | 50 | Hypothetical protein (*L. plantarum*) | (98%) 48/49 | WP_015063551 | - | - |
|  |  |  |  |  |  |  |  |  |  |

ªWithout stop codon.

^b^aa, amino acids.

^c^ORFs annotated during completion and revision of the plasmid sequence and pseudogenes do not have locus_tag or protein_id in GenBank.
