# Supplementary material for: Genome Analysis of Lactobacillus plantarum LL441 and Genetic Characterisation of the Locus for the Lantibiotic Plantaricin C
Source: Front Microbiol. 2018 Aug 17;9:1916. doi: 10.3389/fmicb.2018.01916 (PMC6107846; doi:10.3389/fmicb.2018.01916)
Supplement: Supplementary file 4 [file Presentation_1.pptx]

## Slide 1
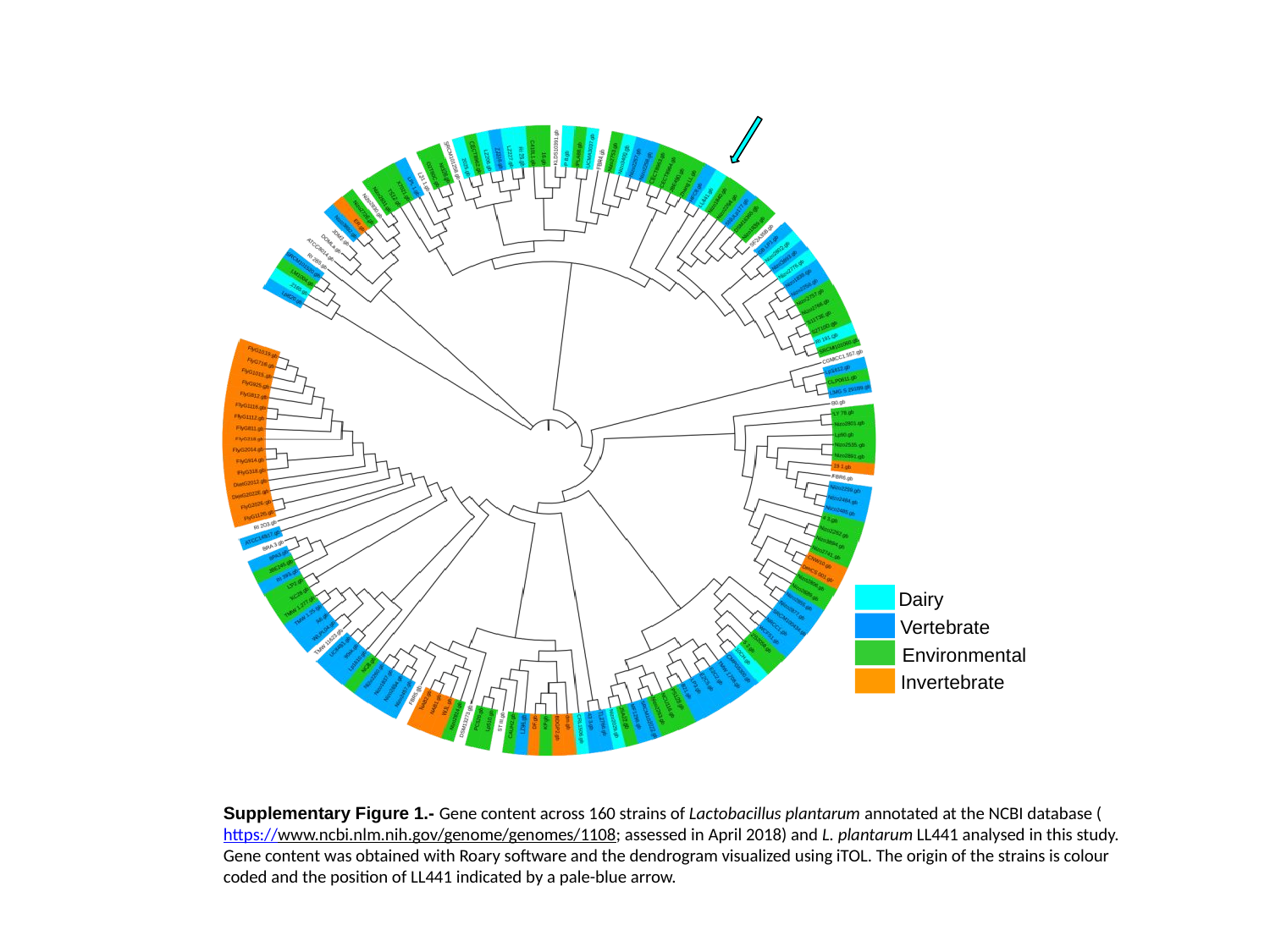

Dairy
Vertebrate
Environmental
Invertebrate
Supplementary Figure 1.- Gene content across 160 strains of Lactobacillus plantarum annotated at the NCBI database (https://www.ncbi.nlm.nih.gov/genome/genomes/1108; assessed in April 2018) and L. plantarum LL441 analysed in this study. Gene content was obtained with Roary software and the dendrogram visualized using iTOL. The origin of the strains is colour coded and the position of LL441 indicated by a pale-blue arrow.
