# Supplementary material for: Genome Analysis of Lactobacillus plantarum LL441 and Genetic Characterisation of the Locus for the Lantibiotic Plantaricin C
Source: Front Microbiol. 2018 Aug 17;9:1916. doi: 10.3389/fmicb.2018.01916 (PMC6107846; doi:10.3389/fmicb.2018.01916)
Supplement: Supplementary file 5 [file Presentation_2.PPTX]

## Slide 1
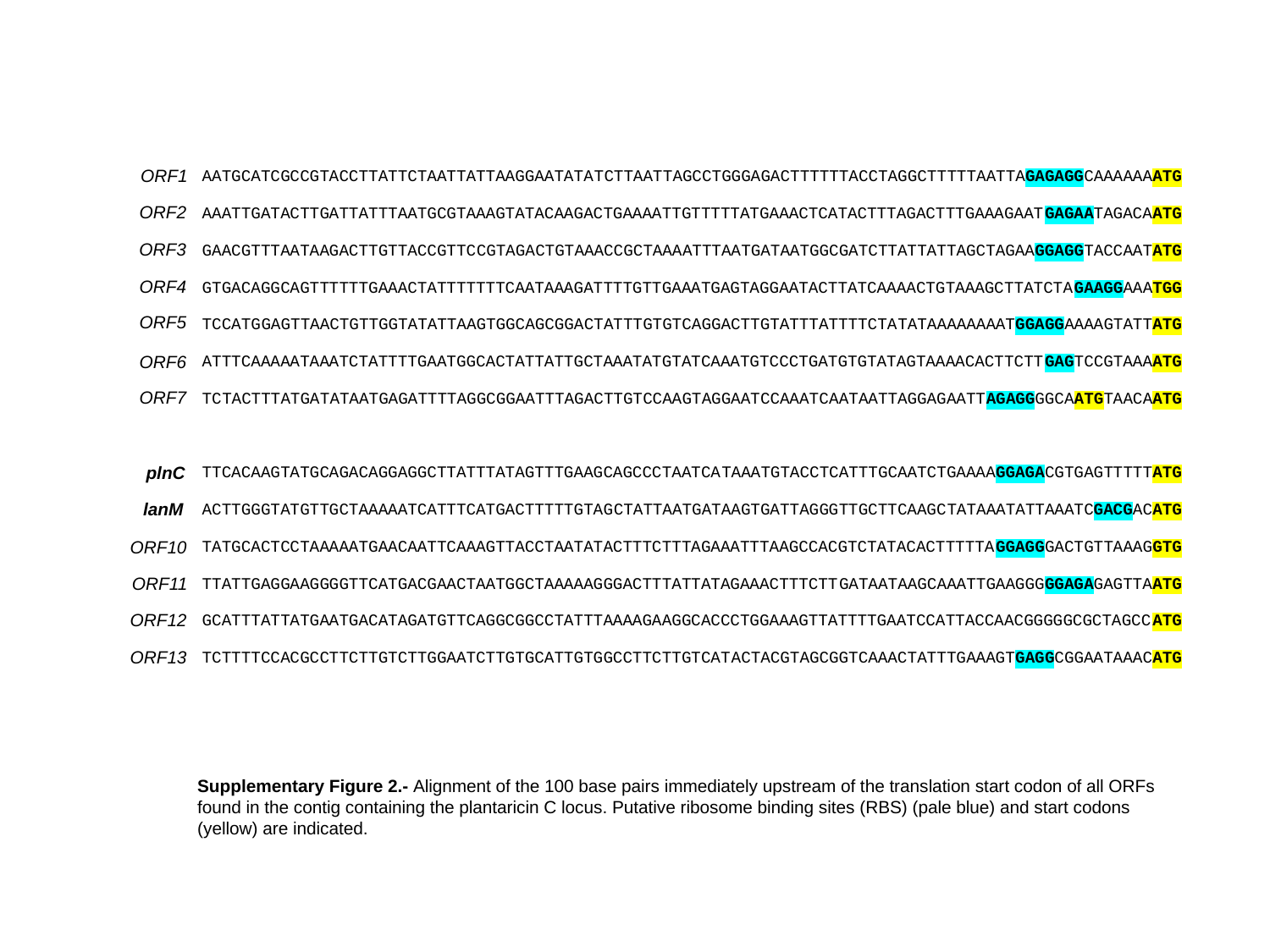

ORF1
ORF2
ORF3
ORF4
ORF5
ORF6
ORF7
plnC
lanM
ORF10
ORF11
ORF12
ORF13
Supplementary Figure 2.- Alignment of the 100 base pairs immediately upstream of the translation start codon of all ORFs found in the contig containing the plantaricin C locus. Putative ribosome binding sites (RBS) (pale blue) and start codons (yellow) are indicated.
