# Supplementary material for: Genome Analysis of Lactobacillus plantarum LL441 and Genetic Characterisation of the Locus for the Lantibiotic Plantaricin C
Source: Front Microbiol. 2018 Aug 17;9:1916. doi: 10.3389/fmicb.2018.01916 (PMC6107846; doi:10.3389/fmicb.2018.01916)
Supplement: Supplementary file 6 [file Presentation_3.PPTX]

## Slide 1
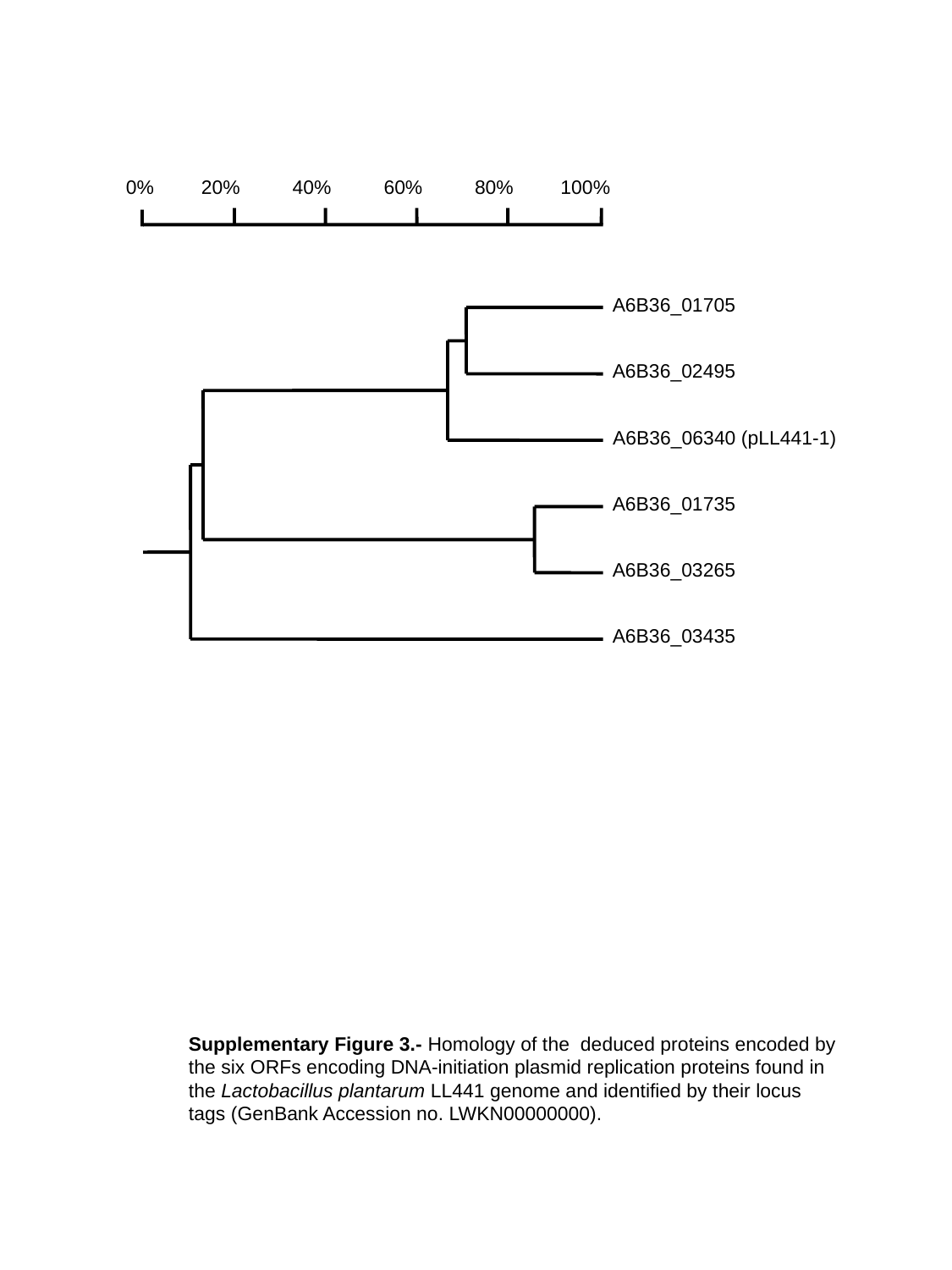

0%
20%
40%
60%
80%
100%
A6B36_01705
A6B36_02495
A6B36_06340 (pLL441-1)
A6B36_01735
A6B36_03265
A6B36_03435
Supplementary Figure 3.- Homology of the deduced proteins encoded by the six ORFs encoding DNA-initiation plasmid replication proteins found in the Lactobacillus plantarum LL441 genome and identified by their locus tags (GenBank Accession no. LWKN00000000).
